# Supplementary material for: Multi-Omics Analyses to Identify FCGBP as a Potential Predictor in Head and Neck Squamous Cell Carcinoma
Source: Diagnostics (Basel). 2022 May 9;12(5):1178. doi: 10.3390/diagnostics12051178 (PMC9140089; doi:10.3390/diagnostics12051178)
Supplement: Supplementary file 1 [file diagnostics-12-01178-s001.zip › diagnostics-1671592-supplementary.pdf]

Supplementary documentation

Supplementary Tables

Table S1. Correlations between *FCGBP* alterations and top altered genes in HNSC patients (n = 522)

A Gene Symbol	B Gene Symbol	Neither	A Not B	B Not A	Both	Log2 (OR)	*q*-Value	Tendency
TP53	*CDKN2A*	112	139	44	214	1.970	< 0.001	Co-occurrence
CDKN2A	*FAT1*	201	165	50	93	1.180	< 0.001	Co-occurrence
TP53	*FAT1*	126	240	30	113	0.984	0.009	Co-occurrence
TTN	*SI*	253	167	39	50	0.958	0.012	Co-occurrence
FCGBP	*FAT1*	354	12	129	14	1.679	0.012	Co-occurrence
FCGBP	*SI*	404	16	79	10	1.676	0.019	Co-occurrence
TP53	*TTN*	102	190	54	163	0.696	0.020	Co-occurrence
FCGBP	*TP53*	153	3	330	23	1.830	0.037	Co-occurrence

HNSC: head-neck squamous cell carcinoma; ***TP53*:** tumor protein p53; ***CDKN2A*:** cyclin-dependent kinase inhibitor; ***TTN***: titin; ***SI***: sucrose-isomaltase; ***FCGBP***: Fc gamma binding protein; **OR**: Odds Ratio

Table S2. Intersection analysis of interacted proteins with FCGBP and *FCGBP*-associated co-expressed genes

Gene Symbol	Combined Score	[1]*p*-Value (co-Expressed Genes)	[2]mRNA Correlation	*p*-Value (mRNA Correlation)
TFF3	0.983	8.27E-17	0.357	3.02E-16
CLCA1	0.789	*	-0.003	*
CDHR5	0.731	*	0.069	*
CHURC1	0.689	*	0.087	*
NPTX2	0.654	*	0.057	*
PCDH17	0.629	*	0.112	1.32E-02
NAALADL1	0.593	6.29E-08	0.304	6.01E-12
ZG16	0.583	4.72E-03	-0.144	1.40E-03
C1QC	0.574	5.93E-03	0.178	7.40E-05
NPAS4	0.555	*	0.13	3.74E-03

[1]***p* value in co-expressed genes:** *p value* of mRNA correlation between *FCGBP* and *FCGBP*-associated co-expressed genes by LinkedOmics; [2]**mRNA correlation:** R value of Spearman's correlation by TIMER2.0.
*$p > 0.05$

Supplementary Figures

Figure S1.

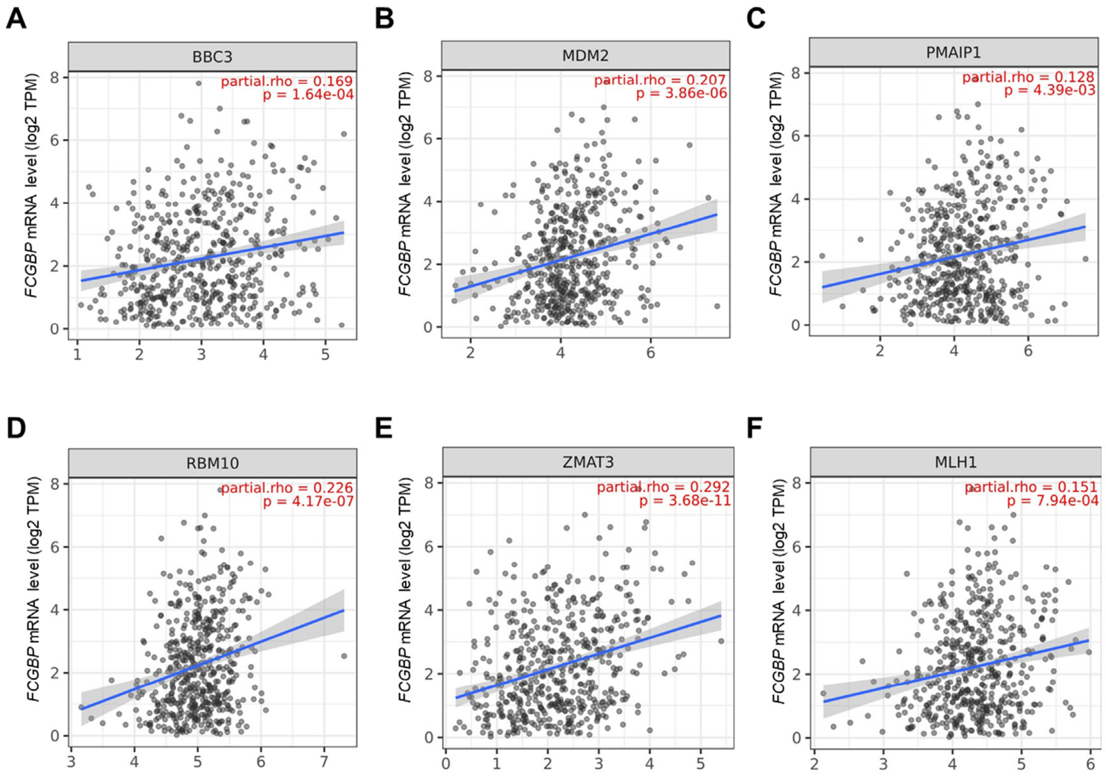

Figure S1. The correlations between *FCGBP* and the target genes of *TP53*.

(A–F) *FCGBP* is positively associated with *BBC3, MDM2, PMAIP1, RBM10, ZMAT3,* and *MLH1*.

Figure S2.

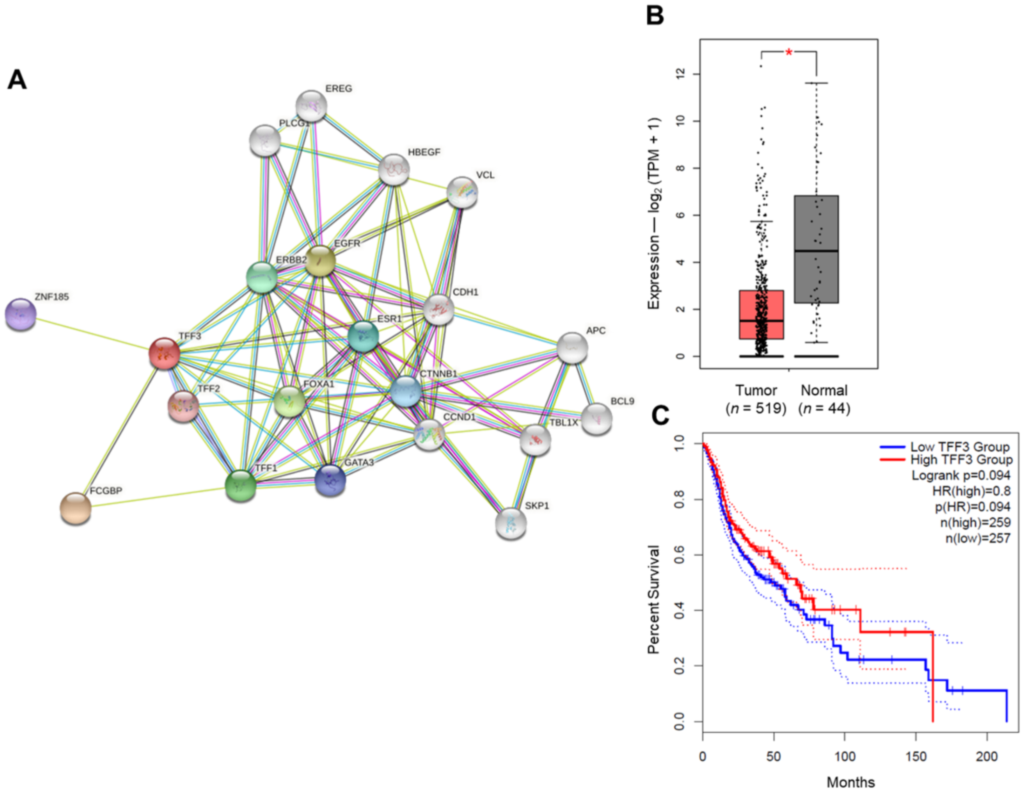

Figure S2. The interacting network, expression levels, and survival analysis of TFF3. (A) Close interaction between TFF3 and EGFR. (B) The expression level of *TFF3* in patients with HNSC (GEPIA2). (C) Kaplan-Meier curve showed overall survival of HNSC patients with high *TFF3* and low *TFF3* levels.

Figure S3.

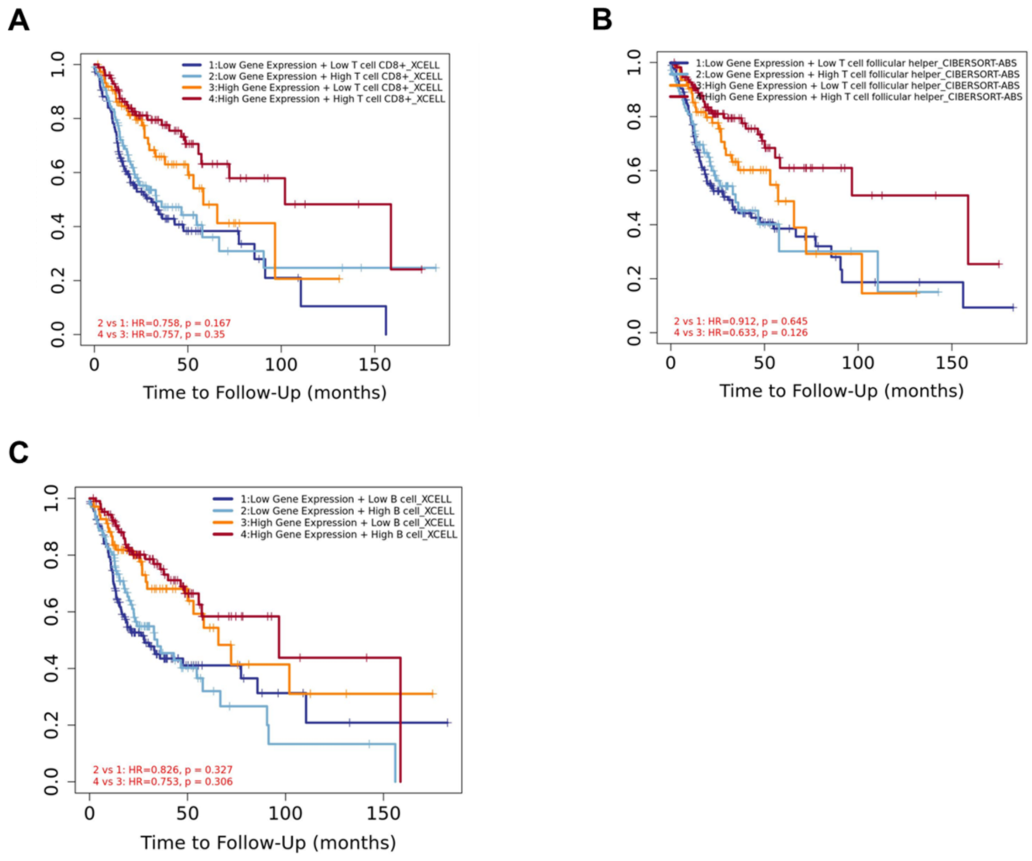

Figure S3. Survival analyses by high- and low- levels of *FCGBP* and infiltration rates of immune cells. (A–C) The prognostic values of infiltration levels of CD8+, follicular helper T cells, and B cells by different *FCGBP* expressions.
